# Gene Therapy Using Plasmid DNA Encoding VEGF164 and FGF2 Genes: A Novel Treatment of Naturally Occurring Tendinitis and Desmitis in Horses

**DOI:** 10.3389/fphar.2018.00978

**Published:** 2018-08-31

**Authors:** Milomir Kovac, Yaroslav A. Litvin, Ruslan O. Aliev, Elena Y. Zakirova, Catrin S. Rutland, Andrey P. Kiyasov, Albert A. Rizvanov

**Affiliations:** ^1^Moscow State Academy of Veterinary Medicine and Biotechnology, Moscow, Russia; ^2^Institute of Fundamental Medicine and Biology, Kazan Federal University, Kazan, Russia; ^3^School of Veterinary Medicine and Science, Faculty of Medicine, University of Nottingham, Nottingham, United Kingdom

**Keywords:** gene therapy, horse, tendon injuries, superficial digital flexor tendon, suspensory ligament, vascular endothelial growth factor 164, fibroblast growth factor

## Abstract

This clinical study describes the intralesional application of the plasmid DNA encoding two therapeutic species-specific growth factors: vascular endothelial growth factor (VEGF164) and fibroblast growth factor 2 (FGF2) in seven horses to restore naturally occurring injuries of the superficial digital flexor tendon (SDFT) (tendinitis) and in three horses with suspensory ligament branch desmitis. Following application all horses were able to commence a more rapid exercise program in comparison to standardized exercise programs. Clinical observation and ultrasonic imaging was used to evaluate the regeneration rate of the tendon and ligament injury recovery and to confirm the safety of this gene therapy in horses, throughout a 12 month period. Follow-up data of the horses revealed a positive outcome including significant ultrasonographic and clinical improvements in 8 out of 10 horses with SDFT and suspensory ligament branch lesions, with return to their pre-injury level of performance by 2–6 months after the completion of treatment. The ninth horse initially presenting with severe suspensory ligament branch desmopathy, showed no significant ultrasonographic improvements in the first 2 months after treatment, however, it improved clinically and became less lame. The final horse, presenting with severe tendinitis of the SDFT returned to their pre-injury level of performance, but experienced re-injury 6 months after treatment. This data is highly promising, however, further research in experimental models, with the histopathological, immunohistochemical and gene expression evaluation of the equine tendon/ligament after gene therapy application is required in order to fully understand the mechanisms of action. This treatment and the significant clinical impacts observed represents an important advancement in the field of medicine.

## Introduction

The most common causes of orthopedic disorders in horses are tendon and ligament injuries and are regarded as a career-limiting disease. It has been estimated that on United Kingdom racecourses for example 46% of all injuries that occur are tendon or ligament injuries (Pinchbeck et al., 2004). Injuries of the SDFT and the SLB are of utmost clinical importance in the horse of all cases observed in equine practice (Kovac et al., 2002).

The healing response following equine tendon/ligament (T/L) injuries is predicable. The response has traditionally been divided into three overlapping stages classified as – inflammation, proliferation/repair and remodeling. The acute inflammatory phase (lasting <10 days) includes phagocytosis of damaged tendon tissue and demarcation of injured tendon tissue. Between 2 and 4 days after injury the proliferative or reparative phase begins, and can last for approximately 45 days. The main problem observed is that at in early stages of tendon healing, increased amounts of collagen type III are observed in the matrix which is synthesized by tenocytes (Juneja et al., 2013). Type III collagen is composed of smaller sized fibers in comparison to type III and has a decreased capacity for elasticity and strength (Sodersten et al., 2013). The collagen fibrils at approximately 45 days after injury are organized into tendon bundles during the remodeling phase, which in itself is often divided into consolidation and maturation phases. During the remodeling phase tenocytes and collagen fibers become aligned in the direction of stress. A decrease in collagen type III is observed with a concomitant increase in type I, and decreases are also observed in cellularity and glycosaminoglycan contents (Sharma and Maffulli, 2006).

Because of the high incidence of equine T/L injuries, high re-injury rate and prolonged recovery period, usually lasting several months and extending up to 15 months in severe injuries, these disorders pose a real problem. Over the years a number of advances in physiotherapy, medical, regenerative and surgical interventions have been developed and applied, however, these strategies are often not sufficient in restoring the functional, structural and biochemical properties of repaired equine T/L to those observed in native tissue. In many cases the repaired tendon tissue remains biochemically and ultrastructurally abnormal even after 12 months and does not completely regain the biomechanical properties it had prior to injury (Yang et al., 2013). When using medicinal therapy to treat equine tendon injuries the disease relapses in more than 43% of cases upon intensive physical exercise (Dyson, 2004). Despite the promise of regenerative medicine methods, such as the use of autologous bone marrow and adipose tissue derived mesenchymal stem cells, as well as platelet rich plasma (Godwin et al., 2012; Middleton et al., 2012; Uysal et al., 2012; Kovac et al., 2016; Witte et al., 2016) which continued to grow since their inception over 10 years ago, translation of experimental models research into clinical therapies by severe equine T/L damage has been frustratingly slow.

The equine tendon is a relatively poorly vascularized tissue. During tendon injury, which is characterized by focal hypocellularity, collagen fibril degeneration, ischemia and fibroblast anoxia, the vascular system is required in order to allow cell infiltration which in turn provides the necessary reparative factors for tissue healing (Docheva et al., 2015). Tenocytes and their extracellular matrix in the form of subunits called fascicles, are surrounded by small amounts of loose connective tissue termed the endotenon. The endotenon carries blood vessels, which do not penetrate the fascicular substance under normal circumstances (Fenwick et al., 2002). The process of angiogenesis is controlled by a variety of mitogenic, chemotactic, or inhibitory peptides and lipid factors (Snedeker and Foolen, 2017). One of the aims of our study was to correct this fundamental problem in horses by stimulating neovascularization/angiogenesis, thus providing the necessary reparative factors during the early tendon healing period. To this end a gene therapy approach using VEGF164 and FGF2 was utilized.

It has been suggested that the ‘use of recombinant proteins and gene therapy are the most advanced and promising approaches in the treatment of musculoskeletal disorders in human medicine’ (Martinek et al., 2005). Angiogenesis is indispensable for regeneration tissue therefore targeted manipulation of this network may offer unique opportunities for regenerative veterinary medicine. We used plasmid DNA containing FGF2 and VEGF164 as they are known to promote soft tissue regeneration. FGF plays minor roles in stages inflammation and remodeling but major roles in stage proliferation/repair and induces VEGF expression – a pivotal factor in the regulation of normal vasculogenesis and angiogenesis (Seghezzi et al., 1998). Several studies have also shown that VEGF can increase the efficiency of skeletal muscle repair by increasing angiogenesis and, at the same time, reducing the accumulation of fibrosis (Best et al., 2013). The plasmid construct pBUDK-ecVEGF164-ecFGF2 was generated as previously described on a pBudCE4.1 vector (Litvin et al., 2016).

The gene therapy approach we recently used in two horses that had gone lame due to injury of the suspensory ligament branch and SDFT resulted in rapid recovery within 2 to 3 weeks (Kovac et al., 2017). Within just 3 months they were back to full health, galloping and competing. The aim of the present study was to provide further clinical proof that a single intralesional injection of plasmid DNA containing species-specific VEGF164 and FGF2 cDNAs has long term clinical and ultrasonographic detectable effect in the treatment of acute and subacute injury of the SLB and SDFT. Clinical assessment and ultrasonic imaging at 12-month follow-up was used in a greater number of horses (10 in total) to evaluate the regeneration rate of the tendon and ligament, and to confirm the safety of this gene therapy. In addition CDU was utilized in order to understand the vasculature of the affected regions in order to further understand the mechanism of action of the gene therapy.

## Materials and Methods

### Study Design

Ten horses with naturally occurring SDFTs and SLB lesions were enrolled onto the study from 2015 to 2017 and treated at the Equine Clinic – New Century of the Moscow State Academy of Veterinary Medicine and Biotechnology, Moscow. The study included seven horses with middle and severe SDFT tendinitis and three horses with desmitis of the SLBs. The horses enrolled on this study were required to meet the following criteria prior to enrollment: acute and subacute tendinitis of SDFT or SLB desmitis, which showed ultrasonographically recognizable hypoechoic lesion occupying >12% of the CSA at the maximum injury zone within an intact paratenon, and a severity of abnormal echogenicity and fiber alignment in which both grades were 3 or higher on a scale from 0 to 4. In addition horses were only included if the clients provided informed consent and if horses had not receive previous intra-tendinous injections.

The owner reported duration from horse injury until initial examination and treatment within the equine clinic was 16.05 days (±14.23), and ranged from 3 to 45 days (**Table 1**). All horses were adults from the following breeds: 3 Hanoverian, 2 Trakehner, 1 Dutch Warmblood, 1 Andalusian, 1 Russian Saddle Horse, 1 Orlov Trotter and 1 Budyonny Horse. The study included 6 gelder, 2 mares and 2 stallions. The average age of the horses was 9.8 years (±2.78), and ranged from 6 to 15 years. The horses involved were mainly used for dressage (8 cases) but one was used for jumping and the other was a pleasure/riding horse (**Table 1**).

**Table 1:** Description, clinical history and diagnostic data of 10 horses before treatment with plasmid DNA encoding VEGF164 and FGF2 genes.

Horse no.	Gender	Breed	Age (years)	Discipline	Reported duration injury until initial examination (days)	Veterinary diagnosis	Injury zone of affected limb and lesion type	Lameness grade before treatment
1	Gelding	Hanoverian	13	Dressage	7 days	Suspensory ligament branch desmopathy	RF 3b lat, Marginal	3
2	Gelding	Andalusian	11	Dressage	14 days	Suspensory ligament branch desmopathy	LF 3b med, Marginal	2
3	Gelding	Hanoverian	9	Dressage	25 days	Suspensory ligament branch and body desmopathy	LH 3a-4b med, Diffuse	3
4	Mare	Dutch Warmblood	7	Dressage	32 days	SDFT tendinitis	LF 2b, Marginal	1
5	Gelding	Trakehner	11	Jumper	3 days	SDFT tendinitis	RF 1b, Marginal	2
6	Gelding	Orlov Trotter	8	Pleasure	12 days	SDFT tendinitis	LF 1b, Diffuse	3
7	Stallion	Trakehner	6	Dressage	45 days	SDFT tendinitis	LF 1a-2a, Core	2
8	Gelding	Russian Saddle Horse	10	Dressage	10 days	SDFT tendinitis and annular ligament desmopathy	RF 3c, Marginal	2
9	Mare	Budyonny Horse	8	Dressage	4 days	SDFT tendinitis	RF 1b, Marginal	2
10	Stallion	Hanoverian	15	Dressage	6 days	SDFT tendinitis	RF 2b, Core	1


**RF, right forelimb; LF, left forelimb; RH, right hindlimb; LH, left hindlimb; SDFT, superficial digital flexor tendon.**

Prior to treatment, all horses underwent clinical examinations (lameness grade), including perineural or intra-articular to precisely localize the lesion. Lameness was evaluated with horses walking and trotting in hand in a straight line, and by lunging on hard and soft surfaces. All the observations and determinations were made by the same veterinarian (M. K.), and were always made on the same ground surface. Lameness diagnosis was performed and scored from 0 to 5 only using the AAEP scale, on which grade 0 represents sound ability and grade 5 indicates non-weight bearing capacity. The signs of inflammation in the palmar metacarpal/plantar metatarsal region (skin surface temperature, swelling, and painful sensitivity to palpation) were also monitored. In these horses with SDFT tendinitis and SLB desmitis, there were no findings of clinical relevance in radiographic examinations in proximal sesamoid bones or any other joints of the lame limb.

### Ultrasonographic Examination

Ultrasonographic examination of the equine T/L was performed using a 7.5-MHz linear-array transducer (Logiq 5, GE Healthcare, WI, United States), after routine clipping. The type of T/L lesion was determined on transverse images in the MIZ (core lesion = centrally located, focal hypo-/anechoic region; marginal lesion = peripherally located, focal hypo-/anechoic region; diffuse lesion = homogenous or heterogenous changes in echogenicity of the whole/most parts of the cross sectional area). A modified ultrasound classification for SDFT was used (Rantanen et al., 2003), with evaluation of the following parameters: total cross-sectional area (T-CSA in mm^2^), total percentage of the cross-sectional lesion area (T-CSA-L %; lesion area/tendon area × 100), T-ES, and the T-FAS. The T-CSA was calculated by summing the CSA of the SDFT in 6 different zones (1A to 3B). The total lesion percentage was calculated as follows: (T-CSA-L/T-CSA) × 100. The severity of the tendon or ligaments lesion was assessed ultrasonographically using the criteria: mild 0–15 of CSA-L %, middle 16–25 of CSA-L % and severe >25 of CSA-L %. Echogenicity was assigned a score of 0 (normoechoic), 1 (hypoechoic), 2 (mixed echogenicity), or 3 (anechoic) and fiber alignment was graded according to the estimated percentage of parallel fiber bundles in the lesion: 0 (>75%), 1 (50–74%), 2 (25–49%), and 3 (<25%). Scores for all levels were summarized to calculate the total echo score and the T-FAS, respectively.

Ultrasound evaluation of the SLB was performed from the palmaromedial and palmarolateral aspects of both limbs. Three equidistant transverse and longitudinal images were obtained for SL branches, and at the level of the sesamoidean insertion. A modified ultrasound evaluation for SLB lesions was used: MIZ-CSA, percentage lesion at the maximum injury zone MIZ, the echogenicity score of the lesion at the MIZ and the percentage disruption of the longitudinal fibers at the MIZ, and also ultrasound evidence of sesamoid bone margin irregularity or disruption at the level of ligament insertion.

The lesion regions of the SDFT and SLB were also evaluated using CDU. Before performing the CDU, the MIZ of the lesion was located with B-mode ultrasonography while the limb was weight-bearing. In order to carry out the CDU, the hoof was placed in a Hickman (Oxspring) block with the digit in slight extension and the carpus flexed at an angle of 80–90°, in order to avoid the possible occlusion of small blood vessels due to mechanical forces. For CDU the settings were established at 10 MHz Doppler frequency, and a pulse repetition frequency of 1.5 kHz. To avoid artifacts, settings were optimized for low flow, and Doppler gain was set just below random noise (VEL/6.2 MHz; 0 Db). Care was taken to minimize pressure exerted by the transducer. The tendons were examined in the longitudinal plane in close proximity to the abnormal areas with a transducer without standoff. The transducer was kept at each site for at least 30 s in lateral, middle, and medial positions, on the tendon to evidence the greatest possible blood flow. The recordings of each CDU session were digitally stored as a videoclip for subsequent DICOM assessment of the vascularity at the end of the study by means of scoring on a semi-quantitative grading scale. On this scale, grade 0 indicated no detectable blood vessels, grade 1 one to two small vessels, grade 2 several small or one to two larger vessels, grade 3 several larger vessels and grade 4 diffuse vascularization, as previously described (Bosch et al., 2011). A clinician blinded to treatment performed the data acquisition, measurements were analyzed and retrospectively for each animal a mean scoring of the lame and also contralateral limb at various time points was produced (R.A).

### Plasmid DNA Drug

Plasmid DNA pBUDK-ecVEGF164-ecFGF2 design was as described previously (Litvin et al., 2016). The plasmid DNA (pDNA) contained coding sequences of *Equus caballus* protein growth factors Vascular endothelial growth factor A 164 (VEGFA164) and fibroblast growth factor 2 (FGF2/bFGF). Plasmid DNA preparation was conducted by GenScript as part of an SC Grade service which guarantees final product purity ≥95% in a supercoiled form with an endotoxin level of ≤0.03 EU/mg pDNA. No additional tests were conducted to verify product purity or percentage present in supercoiled form. VEGF164 and FGF2 were cloned into different expression cassettes under the control of different strong constitutive promoters (Litvin et al., 2016). Our previous work demonstrated high levels of recombinant protein production as shown by immunofluorescence and western blot analysis (Litvin et al., 2016). Descriptions of VEGFA164 and FGF2, alongside the efficiencies of these gene combinations have been shown in our previous work (Plotnikov et al., 2012; Litvin et al., 2016; Kovac et al., 2017).

### Preparation and Administration of the Plasmid DNA

Plasmid DNA preparation, storage and administration was as described previously (Kovac et al., 2017). In short, plasmid DNA was dissolved in 5 ml of a sterile 0.9% NaCl solution to a final concentration of 1 mg/ml. This solution was stored in a fridge at +4°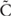 overnight, with occasional gentle stirring and rotation. The pDNA solution was warmed to +37°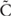 prior to administration into damaged tissue. Before treatment, horses were sedated with detomidine (0.01–0.03 mg/kg intravenously). 3.5 ml total volume of the pDNA solution was drawn into a syringe and under aseptic conditions and sonographic guidance the solution was slowly injected using a 22-gauge needle into sites of injury, whilst the limb was weight-bearing. The filled volume was multi-injected into the most hypoechoic areas of tissue damage and adjacent normal tissues. pDNA was administered on only one occasion per animal in order to treat the tendon and ligament injury.

### Horse Rehabilitation and Evaluation Following Plasmid DNA Gene Therapy

Following injection of the plasmid DNA, a bandage was applied to the limb. Horses were hospitalized for 10 days after procedure for observation and then discharged. Special attention was given to detecting any possible clinical signs of local immunological reactions, including increases in temperature, tissue swelling and lameness. The rehabilitation plan was individually tailored according to the severity of the injury and in relation to results from the clinical and ultrasonographic evaluations throughout the healing process. A controlled exercise program was initiated. We monitored the progress of tendon healing with a modification equine tendon healing criteria according to an already established method (Gillis, 1997), using measurable ultrasound criteria such as the percentage of the cross-sectional lesion, total echo-score and T-FAS. The progress of the SLB healing were also monitored using measurable ultrasound criteria such as the cross sectional area, the percentage lesion at the maximum injury zone, the echogenicity score of the lesion and the fiber alignment score at the maximum injury zone.

The management and rehabilitation program were carefully matched to the progress of healing. As was often noticed in our treated horses, significant clinical and ultrasound improvements were observed at relatively early time periods in comparison to normal tendon healing methods (without gene therapy). In these cases these horses we able to advance more quickly through the exercise program in comparison to those usually prescribed for traditional treatments, this was also in line with previously established techniques (Bosch et al., 2011; Watts et al., 2011).

After stall rest for the first 2 days after treatment, the animals were maintained at a low-level of exercise activity (hand walking) for a 3-week period. Then, when symptom-free, after a warm-up walk, most of the horses trotted for 1–3 min on firm flat ground mainly in straight lines, increasing intensity and time duration every week until entering a complete training protocol including walking, trotting and galloping. Each component of the exercise program was shortened or lengthened on the basis of ultrasonography evidence of healing progression (i.e., healing of the defected tissue) and severity of lameness. The specific exercise program 20 weeks after the administration plasmid DNA was usually mostly determined by the owner/trainer to reflect the normal workload of the horse.

Serial clinic and ultrasound examinations to evaluate the regeneration rate of the T/L injury recovery and to confirm the safety of this gene therapy in horses, were performed for all treated horses: before treatment (day 0), every 20 days in the first 2 months after treatment, then every 30 days in months 3–4 and then every 60 days until the end of the study. At the 12-month follow-up, lameness grade, the time required to return to pre-injury activity level and ultrasonography were also re-evaluated. The treatment was considered to have been successful when the horse returned to its pre-injury training or competition level, without a relapse of the injury during the follow-up time.

### Statistical Analysis

Analysis of data was performed using SPSS 15.0. Quantitative data were evaluated by paired *T*-test. The level of significance was considered as *p* < 0.05. All values in the graphs are expressed as arithmetic mean values with standard deviations.

### Ethics

Plasmid vectors were created in accordance with the human standards set by the United States Food and Drug Administration (FDA, 2007, 2008), and the Committee for the Medicinal Products for Human Use in the European Medicines Agency (EMA, 2001, 2006, 2007). The Institutional Review Board of the Kazan Federal University approved this study (protocol No. 3; date 05.05.2015) in addition to the institutional committees of Moscow State Academy and the University of Nottingham, all national guidelines were adhered to. The horses presented at the clinic with naturally occurring injuries and informed consent was given by owners. This technique has been previously successfully used in dogs (Zakirova et al., 2014) and humans (Plotnikov et al., 2012). Injections and care were given in accordance with standard veterinary practice recommendations and undertaken by qualified clinicians with additional health and welfare checks and clinical observations. Anatomical nomenclature followed the *Nomina Anatomica Veterinaria* 2017 (ICVGAN, 2017).

## Results

### Evaluation of Horses With SLB-Desmopathy

In horses with SLB, desmitis presented with marginal lesions (two limbs) and diffuse lesion (one limbs). Prior to treatment horse #3 had a diffuse lesion not only branch and also moderate damage of the body suspensory ligament of the same leg. In all cases the contralateral limb had either no significant abnormalities on ultrasound. Only horse #1 had ultrasonographic signs of scarring of the SDFT in the contralateral non-lame limb on day 0. Prior to treatment, pain of the ligament by digital pression was noted in all three horses, horses were checked from day 0 until the end of the study. Twenty days after treatment no pain was noted by digital pression at the injury level in horse #1, by day 40 after treatment none of the recorded animals had signs of inflammation in the palmar metacarpal/plantar metatarsal at the lesion region, there were no skin surface temperature changes, swelling, or painful sensitivity to palpation. One horse (#1) returned to pre-injury, sport activity level within 2–6 months of treatment and rehabilitation and s no lameness and participated in competitive dressage tournaments from after the rehabilitation time up to the present day (at the time of manuscript submission). Horse #2 was considered sound following rehabilitation and returned to sporting activity but at a lower level. Horse #3 (SLB) remained lame during the first 3 months of treatment, but there was negligible improvement detected in the degree of lameness and response to the flexion test and after 90 days the lameness was not evident, but this horse not return to sporting activity.

**FIGURE 1:**
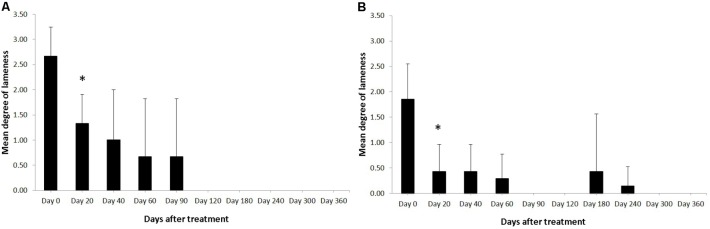
Degree of the lameness in horses with SLB lesions **(A)** and SDFT **(B)** after treatment with plasmid DNA encoding VEGF164 and FGF2 genes. ^∗^Indicates point at which *p* < 0.05 was reached in comparison to day 0.

Horses were observed for adverse reactions from day 0 until the end of the 12 month study. In 2 of the 3 enrolled horses no adverse reactions were observed as a result of the application of the pDNA. Horse #2 showed a minor adverse reaction on the 5th day following administration of the pDNA. The reaction presented as a painless edema and thickening of the subcutaneous tissue in the tissue surrounding the injection point, the edema and swelling gradually disappeared within 10 days. Prior to treatment, on day 0, the mean degree of lameness was 2.67 ± 0.58 in the study horses (**Figure 1A**). In particular horses #1 and #3 showed high degrees of lameness before treatment (**Table 2**). Compared to day 0, lameness significantly decreased by day 20 after treatment to 1.33 ± 0.58 (*p* < 0.05; **Figure 1**). At 12 weeks and persistently in later follow-up examinations, no lameness was evident by clinical examination in any of the three treated horses. The owners were asked to score their horses performance 12 months after gene therapy. Athletic success was reported as good to excellent by 2 out of 3 owners. The owners of horse #3 (the horse that showed lameness within the first 3 months) could not report good-excellent athletic success at that point. The age of the horses and duration of lameness did not show any influence on the clinical outcome after gene therapy. The main differences in the clinical outcomes were observed in relation to the degree and location of T/L damage before treatment.

**Table 2:** Degree of the lameness after treatment of SLB and SDFT over time.

Horse number/days after treatment	SLB				SDFT							
		
	1	2	3	Mean ± *SD*	4	5	6	7	8	9	10	Mean ± *SD*
Day 0	3	2	3	2.67 ± 0.58	1	2	3	2	2	2	1	1.86 ± 0.69
Day 20	1	1	2	1.33 ± 0.58	0	1	1	0	0	1	0	0.43 ± 0.53
Day 40	1	0	2	1.00 ± 1	0	1	1	0	0	1	0	0.43 ± 0.53
Day 60	0	0	2	0.67 ± 1.15	0	1	1	0	0	0	0	0.29 ± 0.49
Day 90	0	0	2	0.67 ± 1.15	0	0	0	0	0	0	0	0 ± 0
Day 120	0	0	0	0 ± 0	0	0	0	0	0	0	0	0 ± 0
Day 180	0	0	0	0 ± 0	0	3	0	0	0	0	0	0.43 ± 1.13
Day 240	0	0	0	0 ± 0	0	1	0	0	0	0	0	0.14 ± 0.38
Day 300	0	0	0	0 ± 0	0	0	0	0	0	0	0	0 ± 0
Day 360	0	0	0	0 ± 0	0	0	0	0	0	0	0	0 ± 0


**Table 3** shows the ultrasonographic measurements of the three horses with SLB desmopathy before and after treatment with plasmid DNA encoding VEGF164 and FGF2 genes. On the2 genes. On the basis of CSA scale of suspensory ligament branch involved in the injury, horses #1 and #2 before treatment showed moderate marginal an- and hypoechoic areas lesions and disruption of fiber pattern, together with enlargement and periligamentar fibrosis in fore limbs (right for horse #1 and left in horse #2). Only in horse #3 was the damage present in the left hindlimb (**Table 3**). In this horse diffuse altered echogenicity of SLB, poor differentiation of ligament margins, periligamentous echogenic material subcutaneously and also middle lesion of suspensory ligament body were noted. In all three cases with SLB desmitis the contralateral limb had no significant abnormalities upon ultrasound before treatment. **Supplementary Figure S1** shows ultrasound images prior to and following treatment. The mean CSAs of the MIZ for SLB before treatment, was 1.98 cm^2^, and the mean of the MIZ-lesions were 30.44% (these ranged from 25.45 to 37.13%; **Table 3**). The echogenicity scores and the percentage disruption of the longitudinal fibers of the lesion at the maximum injury zone before treatment was on average 3 (**Table 3**).

**Table 3:** Ultrasonographic results in horses with SLB desmitis before and after treatment with plasmid DNA encoding VEGF164 and FGF2 genes.

Day after treatment	US-parameter	Horse 1	Horse 2	Horse 3	Mean ± *SD*
Day 0	MIZ-CSA (cm^2)^	1.92	1.77	2.25	1.98 ± 0.20
	MIZ-lesion %	25.45	28.76	37.13	30.44 ± 4.91
	MIZ-ES	3	3	3	3.00 ± 0.00
	MIZ-FAS	3	3	3	3.00 ± 0.00
Day 20	MIZ-CSA	1.66	1.72	1.85	1.74 ± 0.08
	MIZ-lesion %	18.43	16.67	36.63	23.91 ± 9.02
	MIZ-ES	2	2	3	2.33 ± 0.471
	MIZ-FAS	2	3	3	2.66 ± 0.47
Day 40	MIZ-CSA	1.65	1.56	1.99	1.73 ± 0.18
	MIZ-lesion %	12.36	10.46	26.73	16.51 ± 7.26
	MIZ-ES	1	1	2	1.33 ± 0.47
	MIZ-FAS	2	2	3	2.33 ± 0.47
Day 60	MIZ-CSA	1.53	1.48	1.78	1.59 ± 0.13
	MIZ-lesion %	8.78	10.57	24.33	14.56 ± 6.94
	MIZ-ES	1	1	2	1.33 ± 0.47
	MIZ-FAS	2	2	3	2.33 ± 0.47
Day 90	MIZ-CSA	1.60	1.47	1.86	1.64 ± 0.16
	MIZ-lesion %	3.37	6.68	19.25	9.76 ± 6.84
	MIZ-ES	1	1	2	1.33 ± 0.47
	MIZ-FAS	1	2	3	2.00 ± 0.81
Day 120	MIZ-CSA	1.47	1.57	1.70	1.58 ± 0.09
	MIZ-lesion %	3.57	4.78	15.86	8.07 ± 5.53
	MIZ-ES	1	1	2	1.33 ± 0.47
	MIZ-FAS	1	1	2	1.33 ± 0.47
Day 180	MIZ-CSA	1.40	1.34	1.72	1.48 ± 0.17
	MIZ-lesion %	2.41	3.34	12.89	6.21 ± 4.73
	MIZ-ES	0	1	2	1.00 ± 0.81
	MIZ-FAS	1	1	2	1.33 ± 0.47
Day 240	MIZ-CSA	1.45	1.54	1.72	1.57 ± 0.11
	MIZ-lesion %	3.71	2.08	10.99	5.59 ± 3.87
	MIZ-ES	0	0	1	0.33 ± 0.47
	MIZ-FAS	1	1	2	1.33 ± 0.47
Day 300	MIZ-CSA	1.47	1.59	1.85	1.63 ± 0.15
	MIZ-lesion %	1.67	1.98	8.80	4.15 ± 3.29
	MIZ-ES	0	0	1	0.33 ± 0.47
	MIZ-FAS	0	0	2	0.66 ± 0.94
Day 360	MIZ-CSA	1.52	1.55	1.79	1.62 ± 0.12
	MIZ-lesion %	0.84	1.15	7.96	3.31 ± 3.28
	MIZ-ES	0	0	1	0.33 ± 0.47
	MIZ-FAS	0	0	2	0.66 ± 0.94


**MIZ-CSA, maximal injury zone cross-sectional area; MIZ-lesion %, percentage lesion at the maximum injury zone; MIZ-ES, echogenicity score of the lesion at the maximum injury zone; MIZ-FAS, the fiber alignment score at the maximum injury zone*.*

**FIGURE 2:**
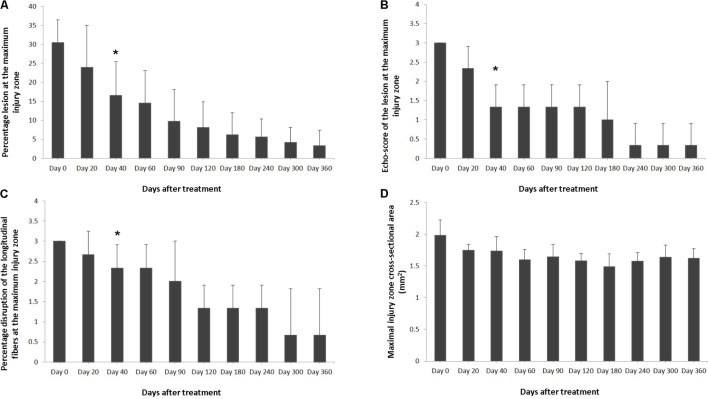
Effects of plasmid DNA encoding VEGF164 and FGF2 genes in horses with SLB desmopathy. The **(A)** percentage of total cross sectional lesion area lesion, **(B)** echogenicity score of the lesion, **(C)** fiber alignment score of the lesion, and **(D)** cross-sectional area of the suspensory ligament branch, at the maximal injury zone. ^∗^ indicates point at which *p* < 0.05 was reached in comparison to day 0.

**FIGURE 3:**
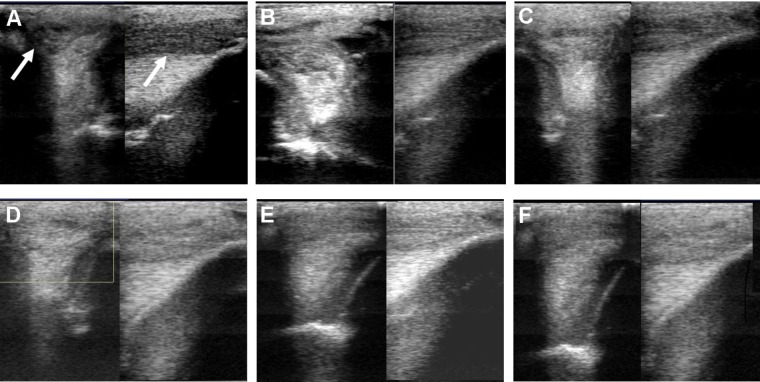
Ultrasound images prior to plasmid DNA encoding VEGF164 and FGF2 genes on day 0 **(A)**, 20 **(B)**, 40 **(C)**, 90 **(D)**, 180 **(E)**, and 300 **(F)** after administration in horse #2 – SLB desmopathy. Arrows indicate lesion.

Ultrasound characteristics of SLB desmopathy in horses #1 and #2 had started to improve at the checks performed 20 days after treatment, and the healing process was constantly maintained in latter follow-ups. This was especially noticeable in the following parameters: MIZ-lesion % (**Figure 2A**), the echogenicity (**Figure 2B**) and the fiber alignment score at the maximum injury zone (**Figure 2C**). Mean MIZ-CSA of the branch of suspensory ligaments did not change significantly throughout the observation period (**Figure 2D**). When these horses started the exercise program, ligament architecture improved constantly, as demonstrated by their longitudinal alignment and length (**Figure 3**). According to our observations, there was no substantial improvement in the first 90 days after treatment in all ultrasonographic parameters in horse #3 with SLB desmitis. On days 20 and 40 this horse showed new hypoechoic lesions, indicating an unstable healing process. Only on days 120–180 after treatment did this horse present with noticeable improvement of the MIZ-lesion %, the echogenicity and the fiber alignment score.

**FIGURE 4:**
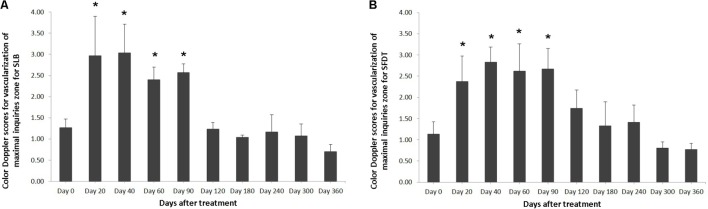
The mean color Doppler scores for vascularization of maximal inquiries zone for **(A)** SLB and **(B)** SDFT after treatment with the plasmid DNA encoding VEGF164 and FGF2 genes. ^∗^ indicates *p* < 0.05 in comparison to day 0.

**FIGURE 5:**
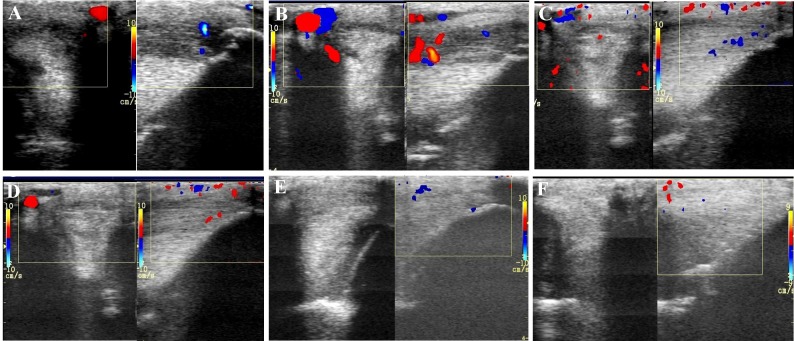
Transverse and longitudinal color Doppler ultrasonography projections prior to plasmid DNA encoding VEGF164 and FGF2 genes **(A)** and 20 **(B)**, 40 **(C)**, 90 **(D)**, 180 **(E)**, 360 **(F)** days after administration in horse #2 – SLB desmopathy.

The evaluated T/L lesions using CDU showed that in most horses with SLB-desmopathy before treatment, only small color foci that were rhythmically blinking at the lesion area were present. The average color Doppler scores for vascularization of maximal inquires zone for SLB before treatment was 1.27 (±0.21), this was slightly elevated in comparison to healthy contralateral limbs (0.8 ± 0.52). Color Doppler scores for vascularization of maximal inquires zone for SLB limbs after treatment with the plasmid DNA encoding VEGF164 and FGF2 genes, revealed a notable increase in scores by just day 20 (2.97 ± 0.93; **Table 4** and **Figures 4**, **5**). The increase continued at day 40 (0.03 ± 0.68), with elevated levels continuing until day 90. The CDU scores in most animals then gradually decreased to basal indices (as in healthy limbs) after 180 days of treatment, indicating blood flow patterns similar to day 0 (**Figure 4**). No significant correlation was observed between the severity of T/L lesions prior treatment and CDU scores after treatment. Horse #2 after 20 days of treatment showed a very high CDU score of 4. This horse, as previously mentioned, had a slight local reaction on days 5–10 at the site of plasmid DNA injection.

**Table 4:** Color Doppler scores for vascularization of maximal inquiries zone for SLB and SDFT after treatment with the plasmid DNA encoding VEGF164 and FGF2 genes.

	SLB				SDFT							
		
Days after treatment/horse number	1	2	3	Mean ± *SD*	4	5	6	7	8	9	10	Mean ± *SD*
Day 0	1.2	1.5	1.1	1.27 ± 0.21	1.1	1.3	0.9	1.0	1.3	1.6	0.7	1.13 ± 0.30
Day 20	2.2	4.0	2.7	2.97 ± 0.93	1.7	2.1	1.8	3.2	2.6	3.1	2.1	2.37 ± 0.60
Day 40	2.5	3.8	2.8	3.03 ± 0.68	2.6	2.7	2.3	2.8	3.1	3.4	2.9	2.83 ± 0.35
Day 60	2.1	2.7	2.4	2.40 ± 0.30	2.8	2.8	1.4	2.1	3.2	2.8	3.2	2.61 ± 0.65
Day 90	2.8	2.4	2.5	2.57 ± 0.21	3.2	3.1	2.8	1.8	2.4	2.5	2.9	2.67 ± 0.48
Day 120	1.2	1.1	1.4	1.23 ± 0.15	1.8	2.1	2.4	1.4	1.1	1.8	1.6	1.74 ± 0.43
Day 180	1.0	1.0	1.1	1.03 ± 0.06	1.2	2.5	1.2	1.2	1.5	0.9	0.8	1.33 ± 0.56
Day 240	0.8	1.1	1.6	1.17 ± 0.40	1.6	1.9	1.7	1.1	0.9	1.0	1.7	1.41 ± 0.40
Day 300	0.9	1.4	0.9	1.07 ± 0.29	0.9	1.0	0.6	0.6	0.8	0.9	0.8	0.80 ± 0.15
Day 360	0.6	0.6	0.9	0.70 ± 0.17	1.0	0.8	0.6	0.8	0.9	0.7	0.6	0.77 ± 0.15


**FIGURE 6:**
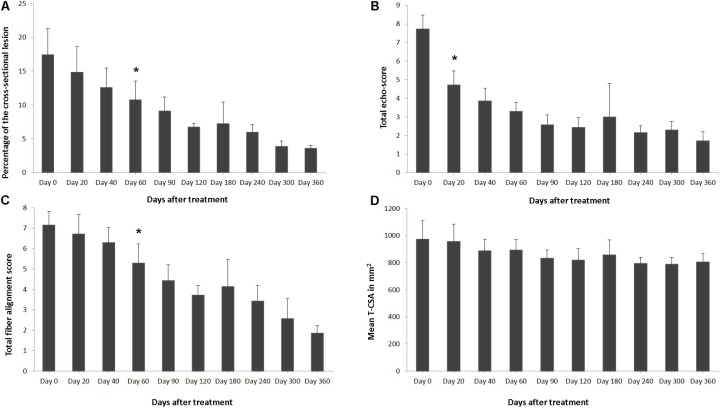
Effects of plasmid DNA encoding VEGF164 and FGF2 genes in horses with SDFT lesions. The **(A)** percentage of total cross sectional lesion area, **(B)** echogenicity score, **(C)** fiber alignment score, and **(D)** cross-sectional area of the suspensory ligament branch at the maximal injury zone. ^∗^ indicates point at which *p* < 0.05 was reached in comparison to day 0.

**FIGURE 7:**
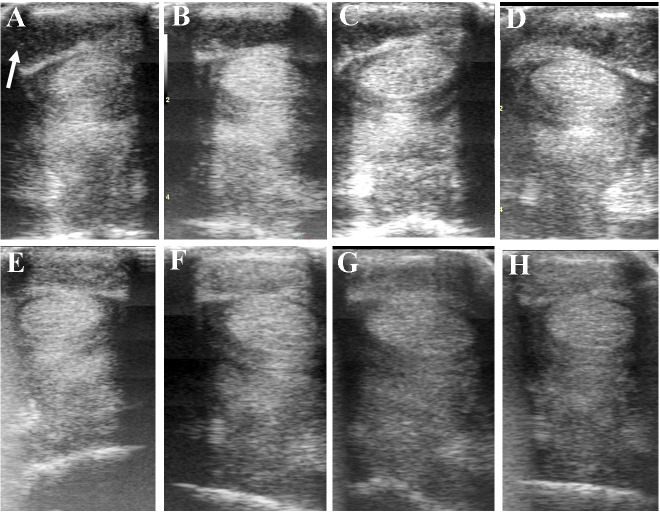
Ultrasound images prior to plasmid DNA encoding VEGF164 and FGF2 genes **(A)** and 20 **(B)**, 40 **(C)**, 60 **(D)**, 120 **(E)**, 180 **(F)**, 240 **(G)**, and 300 **(H)** days after administration in horse #6 – SDFT tendinitis. Arrow indicates lesion.

**FIGURE 8:**
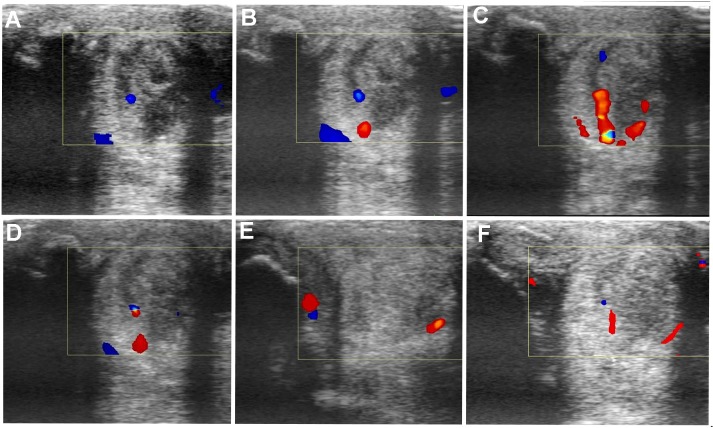
Transverse and longitudinal color Doppler ultrasonography projections prior to plasmid DNA encoding VEGF164 and FGF2 genes **(A)** and 20 **(B)**, 40 **(C)**, 90 **(D)**, 180 **(E)**, 360 **(F)** days after administration in horse #3 – SLB desmopathy.

### Evaluation of Horses With SDFT-Tendinitis

The horses included with SDFT tendinitis presented with core lesions (two limbs), marginal lesions (four limbs) and diffuse lesions (six limbs) (**Table 2**). In horse #8 with acute SDFT lesions in zone 3c, mild damage of the annular ligament and accumulation of the synovial fluid within of the tendon sheath were also detected in the same leg. In all cases the contralateral limb had either no significant abnormalities on ultrasound. Only horses #5 had ultrasonographic signs of scarring of the SDFT in the contralateral non-lame limb on day 0. Prior to treatment, pain of the tendon by digital pression was noted in six of the seven horses, only horse #7 with core lesion of SDFT did not show pain before the treatment. Twenty days after treatment no pain was noted by digital pression at the injury level in six of the seven horses. (horses #4, #5, #7, #8, #9, and #10). By day 40 after treatment none of the recorded animals had signs of inflammation in the palmar metacarpal/plantar metatarsal at the lesion region, there was no skin surface temperature changes, swelling, or painful sensitivity to palpation.

Five horses returned to pre-injury, sport activity level within 2–6 months of treatment and rehabilitation and showed no lameness (horses #4, #5, #7, # 9, and #10). Two of these horses (#4 and #10), participated in competitive dressage tournaments from after the rehabilitation time up to the present day (at the time of manuscript submission). One horse (#8) was considered sound following rehabilitation and returned to sporting activity but at a lower level. Horse #5, with middle acute SDFT tendinopathy, returned to its pre-injury level of performance, but approximately 6 months after the treatment, once competitive jumping had resumed, this animal suffered sudden onset of lameness, which was associated with the recurrent tendon injury in the treated limb. The final horse (Horse #9, SDFT) sustained an unrelated injury on the contralateral limb approximately 11 months after treatment (proximal suspensory ligament desmitis), which precluded analysis of the outcome, but up until that point sporting activities had resumed.

Horses were observed for adverse reactions from day 0 until the end of the 12 months study. There were no adverse reactions in any of the 7 SDFT affected horses as a result of the application of the pDNA. Prior to treatment, on day 0, the mean degree of lameness was 1.86 ± 0.69 in the study horses (**Figure 1B**). In particular horses #6 showed high degrees of lameness before treatment (**Table 2**). Compared to day 0, lameness significantly decreased by day 20 after treatment to 0.43 ± 0.53 (*p* < 0.05; **Figure 1B**). Lameness was eliminated faster in horses with tendinitis of SDFT than horses with desmitis of SLB (*P* > 0.05). At 12 weeks and persistently in later follow-up examinations, no lameness was evident by clinical examination in 6 from 7 treated horses, (horse #5 was affected at days 180 and 240 following recovery at days 90 and 120). The owners were asked to score their horses performance 12 months after gene therapy. Athletic success was reported as good to excellent by six out of seven owners. The owners of horse #5 (the horse with onset of lameness following the healing process) could not report good-excellent athletic success at that point. The age and gender of horses and duration of lameness did not show any influence on the clinical outcome after gene therapy. The main differences in the clinical outcomes were observed in relation to the degree and location of T/L damage before treatment.

**Table 5** and **Figure 6** show ultrasonographic findings of the seven horses with SDFT tendinitis before and after treatment with plasmid DNA encoding VEGF164 and FGF2 genes (see also **Figure 7** and **Supplementary Figure S2**, representative images from horse #6). The average total percentage of CSA scale of SDFT involved in the injury, before treatment was 17.44% (range 12.03–23.38%; **Table 5**). In two patients (horses #7 and #10) the core lesion in SDFT presented with a mixed focal, dishomogeneous, hypo-echoic area. The other horses with acute SDFT tendinitis before treatment presented with marginal (three limbs) and diffuse lesions (two limbs), with loss of the normal fibrillary pattern (**Table 5**). The damage of SDFT was primarily seen on the forelimbs (**Table 5**). The maximum injury zone of most lesions of the SDFT was located in zones 1 and 2 (**Table 5**). Horse #8 prior to treatment presented with acute SDFT lesions in zone 3c, in addition to mild damage and a thickening of the annular ligament and accumulation of the synovial fluid within the fetlock tendon sheath. In all cases of tendinitis of SDFT, the contralateral limb had no significant abnormalities. Horse #9 prior to treatment showed ultrasonographic signs of scarring of the SDFT in the contralateral non-lame limb (day 0). The CSA of the affected SDFT limb at the time of treatment was larger than the CSA of the contralateral normal limb, consistent with injury. The mean T-CSA of the SDFT before treatment was 972.9 mm^2^ (range 867–1272 mm^2^) (**Table 5**).

**Table 5:** Ultrasonographic results of the SDFT tendinitis horse before and after treatment with plasmid DNA encoding VEGF164 and FGF2 genes.

Day after treatment	US -parameter	Horse number							Mean ± *SD*
			
		4	5	6	7	8	9	10	
Day 0	T-CSA	935	1272	913	952	867	981	890	972.90 ± 137.30
	T-CSA-L %	18.21	23.38	15.62	21.03	14.45	12.03	17.35	17.44 ± 3.88
	T-ES	7	9	8	8	7	8	7	7.71 ± 1.29
	T-FAS	8	8	7	7	6	7	7	7.14 ± 0.69
Day 20	T-CSA	954	1232	930	958	889	890	845	956.90 ± 127.80
	CSA-L %	15.35	20.88	14.27	16.45	11.38	9.04	16.58	14.85 ± 3.83
	T-ES	5	5	4	5	4	6	4	4.71 ± 0.75
	T-FAS	8	8	6	7	6	6	6	6.71 ± 0.95
Day 40	T-CSA	879	1055	891	939	812	836	809	888.70 ± 86.89
	T-CSA-L %	12.56	15.67	11.09	14.43	8.65	9.56	15.94	12.56 ± 2.92
	T-ES	4	4	4	3	3	4	5	3.86 ± 0.69
	T-FAS	7	7	6	7	6	5	6	6.29 ± 0.75
Day 60	T-CSA	834	1025	865	822	847	988	865	892.30 ± 80.26
	T-CSA-L %	10.67	14.04	8.99	11.56	8.01	7.56	14.43	10.75 ± 2.76
	T-ES	3	4	3	3	4	3	3	3.28 ± 0.48
	T-FAS	5	6	6	6	6	4	4	5.29 ± 0.95
Day 90	T-CSA	789	918	854	847	767	890	756	831.6 ± 62.34
	T-CSA-L %	8.78	9.18	7.88	12.06	7.05	7.11	11.76	9.11 ± 2.06
	T-ES	3	3	2	3	2	2	3	2.57 ± 0.53
	T-FAS	4	5	5	5	5	4	3	4.43 ± 0.78
Day 120	T-CSA	756	977	803	890	802	764	745	805.30 ± 79.06
	CSA-L %	7.03	7.46	6.55	6.56	6.87	5.75	7.02	6.75 ± 0.54
	T-ES	2	2	3	2	3	3	2	2.42 ± 0.53
	T-FAS	4	4	4	4	4	3	3	3.71 ± 0.48
Day 180	T-CSA	815	1096	868	781	769	823	851	857.60 ± 110.80
	T-CSA-L %	5.85	14.43	5.89	6.60	6.02	5.55	6.31	7.23 ± 3.19
	T-ES	2	7	2	2	3	3	2	3.00 ± 1.80
	T-FAS	4	7	4	4	4	3	3	4.14 ± 1.34
Day 240	T-CSA	811	828	769	853	717	808	776	808.00 ± 44.72
	T-CSA-L %	5.06	7.98	4.97	7.06	5.74	4.88	5.86	5.93 ± 1.17
	T-ES	2	3	2	2	2	2	2	2.14 ± 0.37
	T-FAS	3	5	3	3	3	3	4	3.43 ± 0.78
Day 300	T-CSA	698	786	834	803	765	776	853	787.90 ± 50.57
	T-CSA-L %	3.07	4.99	3.07	4.83	3.08	3.95	4.04	3.86 ± 0.82
	T-ES	2	3	2	2	2	3	2	2.29 ± 0.48
	T-FAS	4	4	2	2	2	2	2	2.57 ± 0.97
Day 360	T-CSA	764	769	801	774	890	747	897	806.00 ± 61.91
	T-CSA-L %	3.99	4.06	3.05	3.55	3.46	3.04	3.88	3.57 ± 0.42
	T-ES	2	2	2	2	2	1	1	1.71 ± 0.48
	T-FAS	2	2	2	2	2	1	2	1.85 ± 0.37


*T-CSA, total tendon cross-sectional area; CSA-L %, percentage of the cross-sectional lesion; T-ES, total echo-score; T-FAS, total fiber alignment score.*

The ultrasound characteristics of SDFT lesions in most horses started to improve at 20 days after treatment. This positive rearrangement trend by SDFT healing process was constantly maintained in later follow-up days. When the horses started their exercise program, tendon architecture showed even better recovery, demonstrated by their longitudinal alignment and length. On 20 day after treatment, it was observed that T-CSA-L in %, T-ES, T-FAS, and T-CSA (**Figures 6A–D**, respectively), were better in all SDFT- patients, with an appreciable tissue filling of lesions, but the degree and rate of change of these ultrasonographic parameters in the further course of time went different ways (**Table 5**). For example, during the observational period of 12 months, it was noticed that there was a reduction in size of the T-CSA (in mm^2^), but the changes were not significant (**Figure 6D**). Compared to the contralateral SDFT limb, T-CSA was not significantly higher during the whole observation period. The T- CSA-L % (**Table 5** and **Figure 6A**) of the SDFT-lesions horses decreased at day 40 after treatment in all horses, except for one horse (horse #9), which showed a higher % CSA-L. Total mean CSA-L in % showed a continuous decrease over time, which was significant (*p* < 0.05) for the first time on day 60, when compared to day 0 (**Figure 6A**). Constant reduction of the total percentage of the cross-sectional SDFT lesion was recorded in later follow-ups at 90, 120, 240, 300, and 360 days. Only on day 180, a mild increase of the percentage of T-CSA-Lesion was observed, however, horse #5 had a recurrence of damage at that point.

The echogenicity score of the SDFT lesion (T-ES) after treatment, decreased continuously and significantly from days 0 to 60 in all horses, except for horse 8, which showed an increase in T-ES between days 40 and 60. The echogenicity score had significantly decreased (*p* < 0.05) by day 20 (**Figure 6B**). Finally, at 3 months after the treatment, the echo-texture in the most horses was more regular and the collagen fibers were most oriented in parallel to the longitudinal axis.

The scores for linear fiber pattern (T-FAS) improved during the course of the study in horses with SDFT lesion, but slowly in comparison with the echogenicity score (**Figure 6C**). T-FAS was significantly (*p* < 0.05) decreased for the first time on day 60. Constant decreases in the total scores for linear fiber patters and echogenicity scores were noticed in later follow-ups study days, but on day 180 a mild increase in these ultrasonographic parameters was observed. This happened concomitantly with the reoccurrence of damage observed in horse #5. After 9 months, signs of tendon lesions could only be detected with difficulty in most SDFT injured horses, as they showed notably correct alignment and a well-organized longitudinal pattern.

The evaluated T/L lesions using CDU showed that in most horses with SDFT tendinopathy before treatment, only small color foci that were rhythmically blinking at the lesion area were present. The average color Doppler scores for vascularization of maximal inquires zone for SDFT and SLB before treatment was 1.13 (±0.30), slightly elevated in comparison to healthy contralateral limbs (0.8 ± 0.52). Color Doppler scores for vascularization of maximal inquires zone for SDFT and SLB after treatment with the plasmid DNA encoding VEGF164 and FGF2 genes, revealed a notable increase in scores by just day 20 (2.37 ± 0.60; **Table 4** and **Figures 4B**, **8**). This trend lasted up to 90–120 days, with a maximum score achieved on day 40 (2.83 ± 0.35). The CDU scores in most animals then gradually decreased to basal indices (as in healthy limbs) after 180 days of treatment, indicating blood flow patterns similar to day 0 (**Figure 4**). No significant correlation was observed between the severity of T/L lesions prior treatment and CDU scores after treatment. There were no significant differences in the CDU scores between the SDFT and the SLB horses, after treatment, therefore the changes in CDU-scores were strictly individual.

## Discussion

Tendon or ligament healing is a long process and is dependent upon the severity and size of the lesion. Various therapies for T/L lesions have been described previously; however, injury recurrence rate is high even after long recovery periods and none of these therapies results in complete tissue regeneration. Previously we demonstrated for the first time that direct gene therapy using injected plasmid DNA encoding species-specific VEGF164 and FGF2 cDNAs resulted in rapid recovery (2–3 weeks) of suspensory ligament branch and SDFT injuries in two horses (Kovac et al., 2017). In the current continuation and extension of the previous study we treated 10 cases in order to restore naturally occurring moderate and severe injuries of the SDFT and branches of the suspensory ligament, with a more rapid exercise program than is traditionally applied in equine T/L lesions (Godwin et al., 2012). According to our clinical observations, the direct gene therapy in 9 out of 10 horses resulted in an earlier reduction of the degree of lameness. The rehabilitation time was also significantly reduced to just 20 weeks which represents just 50% of a usual recovery period for equine tendon/ligament injuries (Reef, 2001). By 2–6 months after the completion of the treatment 9 of the horses showed a successful return to their pre-injury level of sports load, in our cases this was the use of horses in dressage or pleasure/riding. It is important to emphasize that traditionally absolute tissue regeneration is not to be expected even when lameness has subsided following treatment of equine tendinitis. In our study, we observed a rapid regeneration of both the SDFT and the suspensory ligament in most horses within 2–6 months of treatment by measuring the total cross sectional area, percentage of cross sectional area of the lesion, the echogenicity score, and the percentage of parallel collagen fibers. Ultrasonographic characteristics of T/L lesions started to improve notably at 3 weeks. This trend was constantly maintained in later follow-ups. Intensive training of a horse can be integrated into the regime once ultrasound examinations confirm almost complete healing of the injured T/L. When the horses in our trial started the exercise program tendon architecture further improved, which was demonstrated by their longitudinal alignment and length. Partly, these effects could be explained with a coincidence with conditions and phases of normal T/L healing. Nevertheless, our data reflect cases of middle to severe T/L lesions and such values are usually associated with a poor prognosis.

No systemic adverse reactions were observed as a result of the application of the plasmid DNA encoding species-specific VEGF164 and FGF2 cDNAs. The horse that was not responsive to treatment, remained lame during first 3 months after treatment had presented with severe damage to the branch and body of SL. Only one sound horse after rehabilitation (originally presenting with tendinitis of the SDFT) experienced re-injury 6 months after treatment. Twelve months after treatment, 8 out of 10 horse owners judged the outcome of their horses after gene therapy in terms of athletic success as good to excellent. According to our observations, the age and gender of horses and duration of lameness prior to treatment did not show any influence on the clinical outcome after gene therapy. The main factor affecting clinical outcome came from the degree and location of T/L damage before treatment. The data obtained in this clinical study are encouraging and reveal that there could be remarkable positive changes in the early stages of injury healing of both equine tendinitis and desmitis, if defect areas are directly treated with plasmid DNA encoding species-specific VEGF164 and FGF2 cDNAs. Partly, these effects could be explained with a coincidence with conditions and phases of normal tendon healing. Nevertheless, our data reflect only cases of moderate or severe tendon lesions; such values are well known to be associated with a poor prognosis in response to the standard methods of treatment.

A limitation of the present clinical study was the fact that the investigation did not determine the exact mechanism of action of the of direct gene therapy with pBUDK-ecVEGF164-ecFGF2 on healing the injured equine tendons and ligaments. Because of a lack of previous reports on results of treatment in this manner of equine tendinitis and desmitis, we cannot compare our results with results of other treatments. Based on some previous studies, growth factors VEGF and FGF2 are proteins which are primarily released by platelets, fibroblasts and endothelial cells at the site of injury (Byrne et al., 2005). Their mechanisms of action are complex and closely related to other factors of inflammation and regeneration. They are considered to stimulate migration of tendoblasts, fibroblasts and mesenchymal stem cells which are responsible for production of collagen and other constituents of extracellular components of ligaments and tendons such as proteoglycans, glycosaminoglycans and glycoproteins, via enhanced angiogenesis (the process of new blood vessel formation) at a site of injury (Martinek et al., 2005).

An increased number and intensity of the vessels signals in T/L lesion area after treatment detected by the CDU was observed in our study. Evaluated lesions using CDU showed in most horses before treatment, only small color foci that were rhythmically blinking at the lesion area. Color Doppler scores for vascularization of maximal inquires zone revealed a notably increase in scores by just day 20 following treatment. This increase lasted up to 90–120 days, with maximum on day 40. The changes in CDU-scores after treatment were strictly individual. The CDU scores in most our study horses then gradually decreases to basal indices after 180 days of treatment. These changes in CDU scores could be explained, by the fact that normal T/L is hypovascular, and angiogenesis or increased vascularity are associated with an acute injured observed in the proliferative but not in the remodeling phase of the tendon healing (Carvalho et al., 2013), i.e., hypervascularity is normal in the healing process. According our observations there was no significant correlation between severity of T/L lesions prior treatment and CDU scores after treatment. In other words, the positive signals of blood flow might be due to transient increases of blood flows in the inherent vessels in response to hypoxia of tendon tissue associated with injury. According to Hatazoe et al. (2015), a semi-quantitative determination of blood flow in the equine tendon might offer a new method for differentiating between active and inactive inflammation in the tendon associated with the injuries, as well as in deciding the time course of injured tendon bundles. We propose that this effect of angiogenesis is notably enhanced after VEGF164 and FGF2 gene therapy.

We hypothesized that the transfer of FGF2 and VEGF164 genes into equine T/L would augment production of growth factors and collagens that would significantly enhance the healing strength over a critical period of the tendon healing. The FGF2 gene was chosen for a number of reasons. Firstly, FGF is one of main growth factors for tissue repair, and its major action is in the promotion of collagen production, tendon development, proliferation of tenocytes and it also plays a role in promoting the expression of a series of other growth factor genes (Tang et al., 2016). Additionally, FGF is down-regulated during tendon repair under normal circumstances and low levels of FGF may be a principle reason for poor healing potential of the tendon (Tang et al., 2014). The vascular endothelial cell growth factor (VEGF) gene was chosen for a number of reasons. This growth factor is one of the most important angiogenic factors in tendon healing, but under normal circumstances, there are low levels of VEGF expression in the tendon (Berglund et al., 2011). VEGF also enhances collagen production through MAP kinase signaling pathways and increases vascular permeability through the synthesis of endothelial platelet-activating factor (Brkovic and Sirois, 2007). In combination FGF and VEGF increase proliferation and prohibit apoptosis of tendon fibroblasts and effectively correct the insufficiency of the tendon healing capacity (Tang et al., 2016). It has also been suggested that the FGF upregulation may represent an early, sustained signal for angiogenesis (Stavri et al., 1995).

Gene therapy, as one of the most advanced technologies in medicine, is a promising therapy for hereditary diseases and additionally offers novel opportunities for the clinical treatment of numerous orthopedic disorders including injuries of the tendon and ligament (Evans et al., 2009; Bosch et al., 2011; Tang et al., 2016). The use of direct gene therapy with these specific growth factors is also highly promising for the treatment of orthopedic disorders not only in horses but in other animal species and humans (Rizvanov et al., 2018). The successful application of direct gene therapy with a similar plasmid construct based on dog-specific VEGF164 and bone morphogenetic protein (BMP2) genes for the treatment of an injury of the anterior cruciate ligament in a large breed dog has been reported previously (Zakirova et al., 2014). Moreover, in a human clinical case report gene therapy pDNA encoding VEGF and FGF2 genes were used for the treatment of patients with critical lower limb ischemia (Plotnikov et al., 2012). Finally, plasmid DNA pl-VEGF165 (officially registered as Neovasculgen), encoding human VEGF165, demonstrated safety and efficacy in patients with chronic lower limb ischemia for the treatment of atherosclerotic peripheral arterial disease without side effects (Deev et al., 2015, 2017). The high efficacy and safety of the direct pDNA gene therapy has been demonstrated in all of these cases. It should be noted that there are other methods also widely used in the literature. A number of vectors (including plasmid, lentivirus, adenovirus, adeno-associated virus and other modified virus platforms) and delivery systems (including lipofection, cationic nanoparticles, polymers, dendrimers) have been utilized (Dunbar et al., 2018; Rizvanov et al., 2018). Our approach using naked plasmid DNA was considered appropriate as the safest way to administer gene therapy as other approaches can suffer from higher toxicity, immunogenicity and even oncogenic potential (in case of integrating viral vectors). In order to achieve a high efficiency with naked pDNA, several injection points were performed (at a distance of 0.5–1 cm from each other) into both damaged and adjoining normal tissue, to ensure a more complete tissue infiltration. Future trials will include the use of delivery systems in order to ensure optimal efficacy.

Vascular endothelial cell growth factor levels peak following inflammatory reactions and this is especially notable during the proliferative and remodeling phases. FGF2 and VEGF gene therapy *in vitro* and *in vivo* with adeno-associated viral vectors in avian flexor tendons showed increased type I collagen plus other extracellular molecules, accelerated cellular proliferation and improvement in tendon strength and elasticity (Tang et al., 2016).

The clinical and ultrasound data obtained in this study are encouraging but based on a relatively small number of horses, referred for various degrees and locations of T/L lesions. Despite the number of clinical cases, this method of using direct gene therapy for the treatment of T/L injuries in horses is unique and highly promising, however, it requires further research. In order to further assess the direct effects of VEGF and FGF2 gene therapy on the healing of injured tendons and ligaments in horses, evaluation of large numbers of experimental animals with extended follow-up times and randomized controlled clinical trials or a double-blind study is required. A more comprehensive and detailed mechanistic approach would also be obtained by obtaining histological samples and undertaking immunohistochemistry on biopsy materials. Factors such as gene expression levels within the tissues, analysis, identification and quantification of the collagens, functional and intracellular distributions of the proteins and further studies into the biochemistry of the pathological process would all help to ascertain the underlying mechanisms of action. Introducing gene therapy into veterinary medicine clinics is becoming an increasing reality, however, there are a number of challenges which must be solved. This study makes significant advances in the use of gene therapies and in the mechanisms underlying the healing process.

## Author Contributions

MK medical diagnosis, clinical observation of horses, pDNA administration, design of the study (clinical part), and writing the manuscript. YL purification clinical grade pDNA for gene therapy, assistance in pDNA administration, design of the study, collection of data and interpretation of results, and writing the manuscript. RA collection and interpretation of clinical data. EZ testing of pDNA efficiency and editing the manuscript. CR and AK intellectual contribution, analysis of results, and writing the manuscript. AR design of the study, interpretation of results and intellectual contribution into the discussion, and writing the manuscript.

## Conflict of Interest Statement

The authors declare that the research was conducted in the absence of any commercial or financial relationships that could be construed as a potential conflict of interest.

## Supplementary Material

FIGURE S1 Ultrasonography projects prior to plasmid encoding VEGF164 and FGF2 genes in SLB affected horses. Prior to treatment **(A,B)** and post recovery **(C,D)**. Horse #1 **(A,C)** and #3 **(B,D)**.

FIGURE S2 Ultrasound images prior to, and following, plasmid encoding VEGF164 and FGF2 genes in SDFT affected horses. Prior to treatment **(A–E)** and post recovery **(F–J)**. Horse #4 **(A,F)**, #5 **(B,G)**, #7 **(C,H)**, #8 **(D,I)**, and #10 **(E,J)**.

## Abbreviations

AAEP: American Association of Equine Practitioners
CDU: color Doppler ultrasonography
CSA: cross-sectional area
EMA: European Medicines Agency
FDA: food and drug administration
FGF2: fibroblast growth factor 2
LF: left forelimb
LH: left hindlimb
MIZ: maximal injury zone
MIZ-CSA: maximal injury zone cross-sectional area
MIZ-ES: maximal injury zone lesion echogenicity score
MIZ-FAS: maximum injury zone fiber alignment score
MIZ-lesion %: maximal injury zone lesion percentage
RF: right forelimb
RH: right hindlimb
SDFT: superficial digital flexor tendons
SLB: suspensory ligament branches
T/L: tendons/ligaments
T-CSA: total tendon cross-sectional area
T-CSA-L %: total percentage of the cross-sectional lesion area
T-ES: total echogenicity score of the lesion
T-FAS: total fiber alignment score
VEGF164: vascular endothelial growth factor
