# Supplementary figures and images for: Gene Therapy Using Plasmid DNA Encoding VEGF164 and FGF2 Genes: A Novel Treatment of Naturally Occurring Tendinitis and Desmitis in Horses
Source: Front Pharmacol. 2018 Aug 31;9:978. doi: 10.3389/fphar.2018.00978 (PMC6127648; doi:10.3389/fphar.2018.00978)

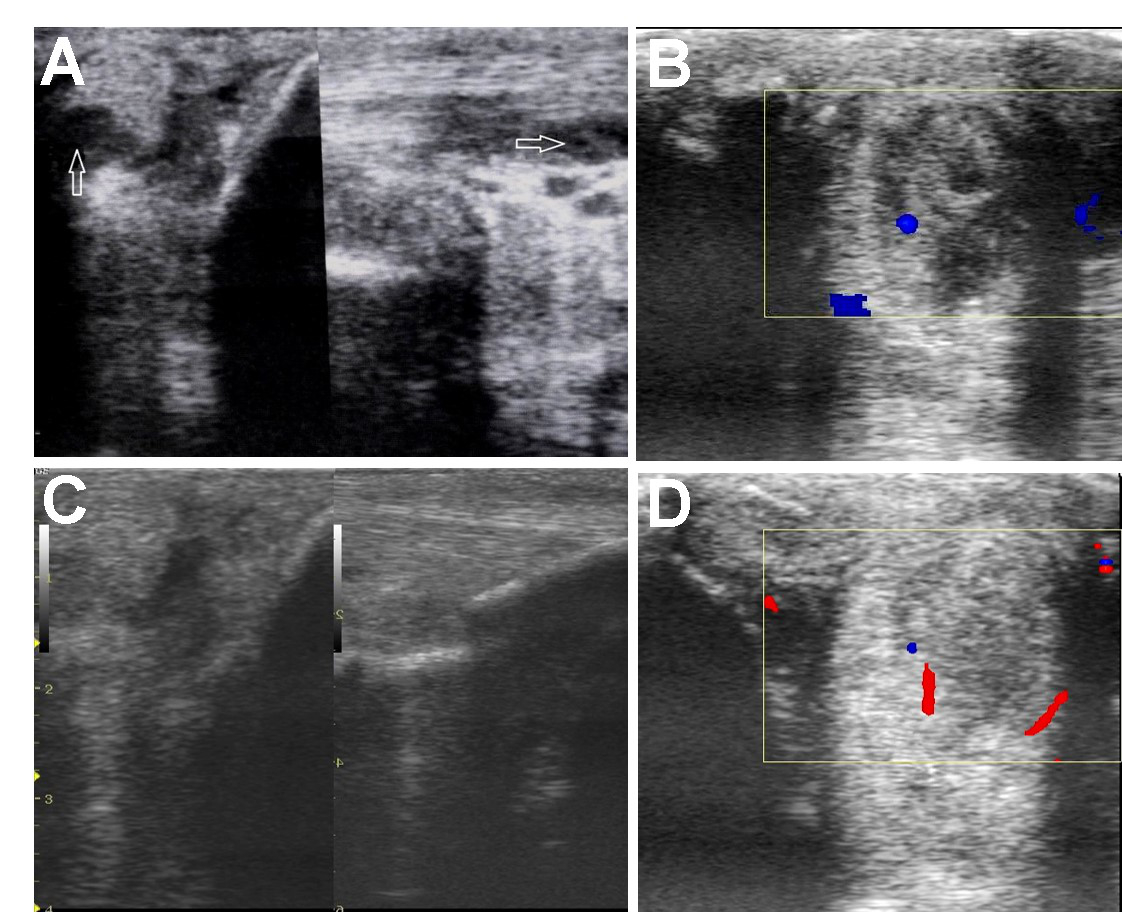

Supplement: FIGURE S1 — Ultrasonography projects prior to plasmid encoding VEGF164 and FGF2 genes in SLB affected horses. Prior to treatment (A,B) and post recovery (C,D). Horse #1 (A,C) and #3 (B,D). [file Image_1.JPEG]

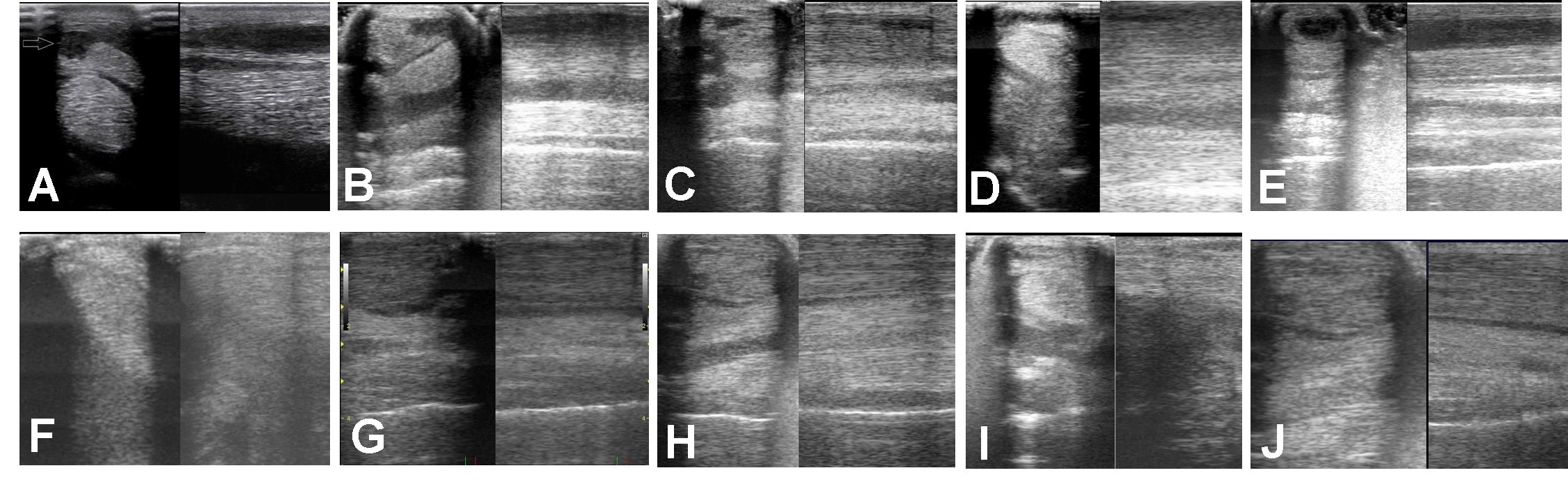

Supplement: FIGURE S2 — Ultrasound images prior to, and following, plasmid encoding VEGF164 and FGF2 genes in SDFT affected horses. Prior to treatment (A–E) and post recovery (F–J). Horse #4 (A,F), #5 (B,G), #7 (C,H), #8 (D,I), and #10 (E,J). [file Image_2.JPEG]
